# Evaluation of the classifications of severity in acute respiratory distress syndrome in childhood by the Berlin Consensus and the Pediatric Acute Lung Injury Consensus Conference

**DOI:** 10.62675/2965-2774.20240229-en

**Published:** 2024-05-20

**Authors:** Roberta Costa Capela, Raquel Belmino de Souza, Maria de Fátima Pombo Sant’Anna, Clemax Couto Sant’Anna

**Affiliations:** 1 Program in Maternal and Child Health Instituto de Puericultura e Pediatria Martagão Gesteira Universidade Federal do Rio de Janeiro Rio de Janeiro RJ Brazil Postgraduate Program in Maternal and Child Health, Instituto de Puericultura e Pediatria Martagão Gesteira, Universidade Federal do Rio de Janeiro - Rio de Janeiro (RJ), Brazil.; 2 Pediatric Intensive Care Unit Instituto de Puericultura e Pediatria Martagão Gesteira Universidade Federal do Rio de Janeiro Rio de Janeiro RJ Brazil Pediatric Intensive Care Unit, Instituto de Puericultura e Pediatria Martagão Gesteira, Universidade Federal do Rio de Janeiro - Rio de Janeiro (RJ), Brazil.

**Keywords:** Severity of Illness Index, Respiratory distress syndrome, newborn, Berlim classification, PALICC classification, Bronchial spasm, Neuromuscular blocking agents, Child

## Abstract

**Objective:**

To compare two methods for defining and classifying the severity of pediatric acute respiratory distress syndrome: the Berlin classification, which uses the relationship between the partial pressure of oxygen and the fraction of inspired oxygen, and the classification of the Pediatric Acute Lung Injury Consensus Conference, which uses the oxygenation index.

**Methods:**

This was a prospective study of patients aged 0 - 18 years with a diagnosis of acute respiratory distress syndrome who were invasively mechanically ventilated and provided one to three arterial blood gas samples, totaling 140 valid measurements. These measures were evaluated for correlation using the Spearman test and agreement using the kappa coefficient between the two classifications, initially using the general population of the study and then subdividing it into patients with and without bronchospasm and those with and without the use of neuromuscular blockers. The effect of these two factors (bronchospasm and neuromuscular blocking agent) separately and together on both classifications was also assessed using two-way analysis of variance.

**Results:**

In the general population, who were 54 patients aged 0 - 18 years a strong negative correlation was found by Spearman’s test (ρ -0.91; p < 0.001), and strong agreement was found by the kappa coefficient (0.62; p < 0.001) in the comparison between Berlin and Pediatric Acute Lung Injury Consensus Conference. In the populations with and without bronchospasm and who did and did not use neuromuscular blockers, the correlation coefficients were similar to those of the general population, though among patients not using neuromuscular blockers, there was greater agreement between the classifications than for patients using neuromuscular blockers (kappa 0.67 *versus* 0.56, p < 0.001 for both). Neuromuscular blockers had a significant effect on the relationship between the partial pressure of oxygen and the fraction of inspired oxygen (analysis of variance; F: 12.9; p < 0.001) and the oxygenation index (analysis of variance; F: 8.3; p = 0.004).

**Conclusion:**

There was a strong correlation and agreement between the two classifications in the general population and in the subgroups studied. Use of neuromuscular blockers had a significant effect on the severity of acute respiratory distress syndrome.

## INTRODUCTION

Acute respiratory distress syndrome (ARDS) manifests as lung inflammation, alveolar edema, and hypoxemic respiratory failure. The pathophysiology of this clinical syndrome is characterized, in succession, by inflammatory, proliferative, and fibrotic phases. Ashbaugh et al. first described ARDS in 1967.^(1)^

Although the first descriptions of ARDS included children, pediatric considerations were not addressed in the American-European Consensus Conference (AECC) or in the Berlin definition. A specific definition for pediatric ARDS was recently put forth by the Pediatric Acute Lung Injury Consensus Conference (PALICC).^(2)^

Adult-based definitions of ARDS may not be applicable to children for a variety of reasons. Anatomical and physiological differences make infants and children more vulnerable to severe respiratory insult than adults, potentially warranting a lower threshold for intervention. Younger patients have a higher metabolic demand and a lower cardiopulmonary reserve than adolescents and adults.^(2)^ Previous applications of adult-based definitions to children, with the need for the measurement of arterial oxygenation, may have led to an underestimated prevalence of pediatric ARDS due to the lesser use of arterial lines in infants and children. Special considerations are also needed to optimize the management of ARDS in the heterogeneous pediatric population, which ranges from neonates to adolescents.

Unlike the Berlin definition (Table 1), the PALICC (Table 2) uses pulse oximetry when the partial pressure of oxygen (PaO_2_) is not available, the oxygenation index (OI) or the oxygen saturation index (OSI) for gravity stratification rather than the relationship between partial pressure of oxygen and the fraction of inspired oxygen (PaO_2_/FiO_2_), and evidence of parenchymal lung disease rather than bilateral radiological infiltrates on chest radiography. Single-center or regional studies have evaluated the PALICC definition, but its performance in large, international samples is unknown.^(3-5)^

**Table 1:** Berlin definition of acute respiratory distress syndrome

Acute respiratory distress syndrome		
Time	Within 7 days of knowledge of the insult or further worsening of respiratory symptoms	
Lung image*	Bilateral opacifications not fully explained by alveolar collapse or nodules	
Origin of edema	Respiratory failure not explained by heart failure or water overload. Need to exclude hydrostatic edema (echocardiogram) if no risk factors are present	
Oxygenation	Lightweight	200mmHg < PaO_2_/FiO_2_ ≤ 300mmHg with PEEP or CPAP ≥ 5cmH_2_O
	Moderate	100mmHg < PaO_2_/FiO_2_ ≤ 200mmHg with PEEP ≥ 5cmH_2_O
	Severe	PaO_2_/FiO_2_ ≤ 100mmHg with PEEP ≥ 5cmH_2_O

Source: Modified from: ARDS Definition Task Force; Ranieri VM, Rubenfeld GD, Thompson BT, Ferguson ND, Caldwell E, Fan E, et al. Acute respiratory distress syndrome: the Berlin Definition. JAMA. 2012;307(23):2526-33.

PaO_2_ - partial pressure of oxygen; F_iO2_ - fraction of inspired oxygen; PEEP - positive end-expiratory pressure; CPAP - continuous airway pressure. * Computed tomography or chest X-ray.

**Table 2:** PALICC definition for acute respiratory distress syndrome in pediatrics

**Pediatric acute respiratory distress syndrome**					
Age	Exclude patients with perinatal lung disease				
Time	Within 7 days of the insult				
Origin of edema	Respiratory failure not explained by heart failure or water overload				
Radiological image	New lung image compatible with parenchymal lung disease				
Oxygenation	Noninvasive mechanical ventilation			Invasive mechanical ventilation	
	PARDS		Lightweight	Moderate	Severe
	Bilevel full-face or CPAP > 5cmH_2_O		4 ≤ OI < 8	8 ≤ OI < 16	OI ≥ 16
	PaO_2_/FiO_2_ < 300mmHg		5 ≤ OSI < 7.5	7.5 ≤ OSI < 12.3	OSI ≥ 12.3
	S/F ≤ 264mmHg				
**Specific groups**					
Cyanotic heart disease		Acute deterioration of oxygenation not explained by the underlying disease			
Chronic lung disease		New infiltrate and acute deterioration of oxygenation according to the above criteria			
Left ventricular dysfunction		New infiltrate and acute deterioration of oxygenation according to the above criteria not explained by left ventricular dysfunction			

Source: Modified from Pediatric Acute Lung Injury Consensus Conference Group. Pediatric acute respiratory distress syndrome: consensus recommendations from the Pediatric Acute Lung Injury Consensus Conference. Pediatr Crit Care Med. 2015;16(5):428-39.

PARDS - acute respiratory distress syndrome in pediatrics; CPAP - continuous airway pressure; OI - oxygenation index; OSI - oxygen saturation index; PaO_2_ - partial pressure of oxygen; FiO_2_ - fraction of inspired oxygen.

In the pediatric intensive care unit (ICU) of our service, two classifications (Berlin and PALICC) have been used for severity stratification of pediatric ARDS since 2015. The overall objective of this study was to classify patients admitted to our pediatric ICU with ARDS according to two severity classifications (Berlin and PALICC). The specific objectives were to describe the profile of patients hospitalized with ARDS at our service, to compare the results of the two severity classifications, and to evaluate the influence of the presence of bronchospasm and the use of neuromuscular blockers (NMBs) in the classification of ARDS severity in an attempt to come to the most appropriate classification of pediatric ARDS for early diagnosis and targeted use of available resources.

## METHODS

This was a prospective, observational study that was conducted at the pediatric ICU of the *Instituto de Puericultura e Pediatria Martagão Gesteira* of the *Universidade Federal do Rio de Janeiro* (UFRJ), from December 2020 to December 2021. This institute is a tertiary pediatric hospital that serves the population of the State of Rio de Janeiro through the Unified Health System (SUS - *Sistema Único de Saúde*). The study was approved by the Research Ethics Committee of the institute (CAAE: 87266418.9.0000.5264).

Children and adolescents aged 0 - 18 years with a diagnosis of ARDS who were invasively mechanically ventilated in assisted-controlled or controlled pressure modes were included. We excluded patients with signs of intracranial hypertension, those who met the criteria for brain death and/or hemodynamic instability, those who were mechanically ventilated invasively in spontaneous mode, and those whose samples no longer met the diagnostic criteria for ARDS during the data collection period (3 days). The sample was enrolled by convenience, comprising all patients admitted to our PICU who met the above criteria in the predetermined 1-year period of data collection.

Data from the medical records were collected at admission and throughout the patient’s stay in the pediatric ICU. Bedside data (both mechanical ventilator data and arterial blood gas analysis) were collected on 3 days during the first week of their ARDS diagnosis: the day of enrollment, 24 hours later, and 48 hours after that.

The severity indices of patients admitted to the PICU were calculated: the Pediatric Index of Mortality (PIM) in the first hour after admission and the Pediatric Risk of Mortality (PRISM) in the first 24 hours after admission.

Sedation was sometimes needed to collect arterial blood gases, as patients on controlled ventilation could have variable peak inspiratory pressure measurements, depending on the degree of agitation, not the pathophysiological characteristics of the disease.

A database was built using Microsoft Excel® version 2013 (Microsoft Corporation, USA). Data analysis was performed using the Statistical Package for Social Sciences software (SPSS, IBM Corporation), version 28.0.0.0. Initially, a descriptive analysis of the data was performed. The qualitative variables are presented as tables of absolute and/or percentage frequencies, and the quantitative variables are presented as measures of central tendency and dispersion: means, standard errors, and medians. For quantitative variables, the normality of the samples was verified using the Kolmogorov‒Smirnov test, with the level of statistical significance ≥ 0.05.

For the comparative analyses between the Berlin (PaO_2_/FiO_2_) and PALLIC (OI) criteria, we quantified the agreement between the severity classifications by the kappa coefficient (κ): very weak agreement was defined as κ from 0 to 0.19; weak agreement, 0.2 to 0.39; moderate agreement, 0.4 to 0.59; strong agreement, 0.6 to 0.79; and very strong agreement, ≥ 0.8.^(6)^ Spearman’s correlation coefficient between the values of the two indices (PaO_2_/FiO_2_ and OI) was calculated: an absolute value of the coefficient between 0 and 0.3 meant negligible correlation; between 0.31 and 0.5, weak; between 0.51 and 0.7, moderate; between 0.71 and 0.9, strong; and > 0.9, very strong.^(7)^ For both tests, a level of statistical significance ≤ 0.05 was considered.

When comparing the two systems, we considered factors that can influence the classification criteria for ARDS. To do this, we separated the patients were separated into two groups, those with and without bronchospasm/lower airways obstructive disease and those with and without the use of NMB, and agreement and correlation were assessed using the κ and Spearman tests, accepting statistical significance at ≤ 0.05.

The influence of the independent variables bronchospasm/lower airway obstructive disease and NMB on Berlin and PALICC scores were evaluated separately and together using two-way analysis of variance (ANOVA) (threshold for significance: p ≤ 0.05). The same procedure was performed for some variables related to respiratory mechanics.

In situations in which means or medians were compared, the t test for independent samples or the Mann‒Whitney test was used, respectively, with statistical significance set at p ≤ 0.05.

## RESULTS

There were 56 patients who were hospitalized with a diagnosis of ARDS, and two were excluded due to refusal of consent. Among the 54 patients in the study, the median age was 30 months, the weight was 10kg, the PIM2 was 5.6%, and the PRISM score was 4.8%. Thirty-two (59.3%) patients were male, and 35 (64.8%) had comorbidities at the time of ARDS diagnosis. Among the admission diagnoses, 34 (63%) patients had pneumonia, 4 (7.4%) had bronchiolitis, and 3 (5.6%) had acute lymphoblastic leukemia. ARDS of pulmonary origin was responsible for 35 (64.8%) patients. The median hospital stay was 12 days (range 4 days to 86 days). The median time on invasive mechanical ventilation was 8 days (range 3 to 66 days). Twenty-one patients had bronchospasm (38.9%).

Fourteen patients died (25.9%) during the study period. Only three patients (5.5%) died within 3 days of data collection, the other deaths occurring later in the pediatric ICU. The first three deaths were of patients with comorbidities such as combined immunodeficiency, biliary atresia, and Burkitt lymphoma that were classified as moderate ARDS progressing to severe ARDS. The causes of death were shock, refractory hypoxemia, and dysfunction of multiple organs and systems.

During the 3 days of data collection, similar ventilatory parameters were used, with a median positive end-expiratory pressure (PEEP) of 8cmH_2_O, FiO_2_ of 60%, peak inspiratory pressure (PIP) of 24cmH_2_O, tidal current (Vt) of 8mL/kg, PaO_2_/FiO_2_ of 145, and OI of 11.

The mechanical ventilation data of the patients included in the study were tested for normality of distribution using the Kolmogorov‒Smirnov test. Only PaO_2_/FiO_2_ presented a normal distribution (p = 0.2).

One to three arterial blood gas samples were collected from the 54 patients within 7 days of ARDS diagnosis. Those with fewer than three samples collected had their samples excluded because they no longer met the classification criteria for ARDS, died, or were changed to spontaneous ventilatory mode during these 3 days. We used the data from up until they were excluded.

There were no criteria for ARDS according to both classifications in 13 samples (8%), according to PALICC in four samples (2.5%) or according to Berlin in two samples (1.2%) over the 3 days, for a total of 19 samples, and there were three deaths in the same period of data collection. Therefore, 22/162 (13.5%) samples were removed from the comparative analyses, leaving 140 valid samples.

In 104 (74.3%) measurements, the patients were evaluated on the Richmond Agitation and Sedation Scale (RASS).^(8)^ With scores of -1 to -5, 69 (49.3%) patients had spontaneous triggering, and 135 (96.4%) had patient-mechanical ventilator synchrony. NMB was used in 72 (51.4%) of the 140 measurements, and there was no associated bronchospasm in 90 (64.3%) measurements. Measurements of ventilatory mechanics were performed in 55 (39.2%) of the total measurements, 42 (30%) of which were measured while the patient was on a NMB. We found a median static compliance of 0.56mL·cmH_2_O ^-1^·kg^-1^, a plateau pressure (Pplat) of 25cmH_2_O and a driving pressure of 14cmH_2_O. Using the 140 measurements of patients diagnosed with ARDS by the two classification criteria over 3 days, the results presented in table 3 were obtained.

**Table 3:** Patients with acute respiratory distress syndrome according to the two classification criteria over the 3 days and agreement between the classifications

**ARDS severity classification**	**Berlin Consensus**	**PALICC**	
Lightweight	25 (17.9)	40 (28.6)	
Moderate	75 (53.6)	57 (40.7)	
Severe	40 (28.6)	43 (30.7)	
Total	140 (100)	140 (100)	
**ARDS severity classification**	**Agreement between the Berlin and PALLIC severity classifications**		
		**Kappa**	**p value**
Lightweight	23 (16.4)	0.62	< 0.001
Moderate	49 (35.0)		
Severe	34 (24.3)		
Total	106 (75.7)		

ARDS - acute respiratory distress syndrome; PALICC - *Pediatric Acute Lung Injury Consensus Conference*. The results are expressed as n (%), when not indicated otherwise.

When the 50 samples from patients with bronchospasm were taken separately, there was agreement between the two ARDS classifications in 38 measurements (76%): 9 samples (23.6%) for mild ARDS, 19 samples (50%) for moderate ARDS, and 10 samples (26.3%) for severe ARDS [κ = 0.62 (p < 0.001)].

For patients without bronchospasm, who gave a total of 90 samples, there was agreement between the two ARDS classifications in 68 measurements (75.5%), 14 samples (20.5%) for mild ARDS, 30 samples (44.1%) for moderate ARDS and 24 samples (35.2%) for severe ARDS [κ = 0.62 (p < 0.001)].

The classification systems were also tested for agreement, according to the use or absence of NMBs, as shown in Table 4. In 60% of the patients with bronchospasm, a NMB was used. The agreement between the classifications for these samples was 76.6%, with a coefficient κ = 0.59. For the samples from patients with bronchospasm without the use of NMB, the agreement between the classifications was similar: 75.5%, with κ = 0.6. There was 71% agreement among patients who used NMBs without bronchospasm, with a coefficient of agreement κ = 0.54. In the samples in which there was neither NMB nor bronchospasm, the agreement between the classifications was 79%, with κ = 0.67. For all participants, the level of statistical significance was p ≤ 0.05.

**Table 4:** Patients with acute respiratory distress syndrome according to both classification criteria, with and without the use of neuromuscular blockers, over the 3 days and agreement between the classifications

**ARDS severity classification (WITHOUT NMB use)**	**Berlin Consensus**	**PALICC**	**Agreement**	
			**Kappa**	**p value**
Lightweight	20 (29.4)	27 (39.7)	0.67	< 0.001
Moderate	34 (50.0)	27 (39.7)		
Severe	14 (20.6)	14 (20.6)		
Total	68 (100)	68 (100)		
**ARDS severity classification (WITH NMB use)**	**Agreement between the Berlin and PALLIC severity classifications**		**Agreement**	
			**Kappa**	**p value**
Lightweight	5 (6.9)	13 (18.0)	0.56	< 0.001
Moderate	41 (56.9)	30 (41.7)		
Severe	26 (36.2)	29 (30.3)		
Total	72 (100)	72 (100)		

ARDS - acute respiratory distress syndrome; NMB - neuromuscular blockade; PALICC - *Pediatric Acute Lung Injury Consensus Conference*. The results are expressed as n (%), when not indicated otherwise.

For the comparison between the values of PaO_2_/FiO_2_ and the OI, all 140 valid samples were used to calculate the correlation between the two variables. Spearman’s correlation coefficient was ρ = -0.918 (p < 0.001), meaning they were negatively correlated. The 50 patients with bronchospasm had a Spearman correlation coefficient ρ = -0.91 (p < 0.001), and the 90 patients without bronchospasm had ρ = -0.91 (p < 0.001). The 72 patients who used NMBs had ρ = -0.92 (p < 0.001), and the 68 patients who did not use NMBs had ρ = -0.9.

Two-way ANOVA was performed to evaluate the effect of the independent variables bronchospasm/lower airway obstruction, NMB use, and the two together on the classification criteria for ARDS (Berlin and PALICC), in addition to other variables related to respiratory mechanics (Table 5). The quantitative variables entered into ANOVA had a normal or homogeneous distribution, as assessed by the Kolmogorov-Smirnov test and the Levene test, respectively (both p > 0.05).

**Table 5:** Comparison between means and medians and influence of the presence of neuromuscular blockers and/or lower airway obstruction (bronchospasm) in patients with criteria for acute respiratory distress syndrome according to the *Pediatric Acute Lung Injury Consensus Conference* and the Berlin Consensus

	NMB	n	Mean ± SD	Median	NMB effect		Obstruction effect GO		NMB effect and GO obstruction	
					F test	p value	F test	p value	F test	p value
PaO_2_/FiO_2_	Yes	72	126.3 ± 52.8	126	12.9	< 0.001	0.029	0.86	7.36	0.39
	No	68	161.7* ± 65.1	163.5						
OI	Yes	72	17.8 ± 15.7	13.4†	8.7	0.004	1.94	0.16	0.04	0.83
	No	68	11.4 ± 8.0	8.7						
C.din (mL.cmH_2_O^-1^.kg^-1^)	Yes	72	0.37 ± 0.14	0.37	23.09	< 0.001	3.84	0.05	5.1	0.03
	No	66	0.51 ± 0.25	0.48†						
C.est (mL.cmH_2_O^-1^.kg^-1^)	Yes	42	0.61 ± 0.36	0.55	0.001	0.98	0.54	0.46	0.61	0.44
	No	13	0.62 ± 0.30	0.62‡						
PIP (cmH_2_O)	Yes	72	27.8 ± 5.2	27.0†	22.1	< 0.001	4.98	0.03	2.3	0.13
	No	68	23.8 ± 6.3	22.0						
Vt	Yes	72	7.9 ± 3.5	8.0‡	0.3	0.58	0.71	0.40	0.004	0.95
	No	68	7.6 ± 2.4	7.0						
MAP (cmH_2_O)	Yes	72	17 ± 3.7	16.5†	12.6	< 0.001	5.3	0.03	0.04	0.95
	No	68	14 ± 3.8	13.8						

NMB - neuromuscular blockade; LAP - lower airways; PaO_2_ - partial pressure of oxygen; FiO_2_ - fraction of inspired oxygen; OI - oxygenation index; Cdin - dynamic compliance; Cest - static compliance; PIP - peak inspiratory pressure; Vt - tidal volume; MAP - mean airway pressure. * Difference in means - t test - p < 0.05; † median difference - U test - p < 0.05 or ‡ p > 0.05.

## DISCUSSION

The profile of pediatric patients admitted to our PICU was consistent with that found in the literature regarding epidemiology, morbidity, and mortality in pediatric ARDS patients.^(8-11)^

The main finding of this study was the significant agreement between two classifications of pediatric ARDS severity (Berlin and PALICC), accompanied by a very strong numerical correlation between PaO_2_/FiO_2_ and OI and the presence of a significant effect of NMB on both classifications.

The presence of bronchospasm, as well as patient–ventilator dyssynchrony, can lead to an increase in PIP due to the increase in resistive pressure and autoPEEP and not elastic pressure, the latter being related to the pathophysiology of ARDS. An increase in PIP leads to an increase in the mean airway pressure (MAP), and with this increase, there is an increase in the OI, which could be interpreted as more severe ARDS than real ARDS.

In patients using NMB or in deep sedation with suppression of spontaneous respiratory triggers, the patient’s effort does not interfere with ventilation, so it is possible to evaluate respiratory mechanics, such as static compliance.^(9)^ On the other hand, suppression of the patient’s respiratory drive and effort leads to increased pleural pressure, necessitating higher distending pressure and PEEP. Therefore, the secondary hypotheses that the presence of bronchospasm and/or the use of NMB could influence the classification criteria for ARDS deserve to be analyzed.

In the present study, there was greater agreement between the classifications in patients who did not receive NMBs, suggesting their influence on the severity of ARDS. For patients with bronchospasm, no such difference was observed. When the four situations with and without bronchospasm/lower airway obstruction and with and without the use of NMB were combined, there was a trend toward an effect of both variables on the ARDS classification. When we tested these effects by two-way ANOVA, the NMB variable had stronger effects on PaO_2_/FiO_2_ and OI. Although there was a significant effect of bronchospasm/lower airway obstruction on PIP, MAP, and dynamic compliance, there was no effect on PaO_2_/F_iO2_ or OI, either independently or together with the NMB variable.

Considering the significant effect of NMBs on the severity classifications (PaO_2_/FiO_2_ and OI) and on PIP and MAP (Figure 5) and the effects of its use in patients with ARDS, the suppression of respiratory drive often causes the need for increased distending pressure and PEEP due to increased pleural pressure, favoring greater collapse, especially in dependent regions. Such effects have been described in patients under deep sedation and maximized in patients using NMB.^(9)^

The increase in pressures required to compensate for the patient’s lack of exertion leads to an increase in MAP, which leads to an increase in OI. As a result, the patient is classified as having ARDS of greater severity when using a NMB. This fact leads to a discussion about the actual severity of ARDS when assessed in the presence of respiratory effort of the patient and how much this can “mask” the severity of ARDS and aid in mechanisms of secondary lung injury.^(10)^

The ARDS is a disease that affects the lung parenchyma and compromises the elastic component of the lung, and PaO_2_/FiO_2_ is one of the most important and sensitive markers of the change in oxygenation related to this syndrome. The difference between the Berlin and PALICC definitions is in the presence of MAP. Since MAP may be higher in patients who use NMBs to compensate for a lack of respiratory drive, perhaps the classification of ARDS severity by PaO_2_/FiO_2_ is more appropriate in these patients.

In the present study, after verifying the effect of NMB on the ARDS classification, we analyzed the behavior of some variables by comparing their medians in situations with and without NMB, including dynamic compliance (C.din = Vt/PIP - total PEEP) and static compliance (C.est = Vt/Pplat - total PEEP). A statistically significant difference was observed only for dynamic compliance, its median value being higher in patients without NMBs. These findings fit the hypothesis that muscular effort affects PIP by generating lower pressures (assuming that patient–ventilator synchrony is present in most patients in the study). No such differences were observed for static compliance, whose calculation does not include respiratory effort. Pplat was used, so only the pulmonary elastic component was evaluated.

In neither situations, the tidal volumes did not show significant differences in their medians, reinforcing the effect of pressure on the observed values for compliance. Similarly, the distributions of patients classified as having moderate to severe ARDS by the two criteria, using or not using NMB, were very similar, providing more robustness to the results described.

The PALICC was created to overcome the limitations of applying adult-based definitions of ARDS to children, including epidemiological differences and the need for invasive measures of oxygenation. The PARDIE study^(11)^ confirmed the better performance of PALICC in terms of identifying new cases of pediatric ARDS and risk stratification. However, in the PARDIE study, fewer than half of the new diagnoses of pediatric ARDS were made by means of invasive oxygenation measurements. In contrast, in our unit, arterial lines are routinely installed at the beginning of mechanical ventilation for better hemodynamic monitoring.

We sought a patient sample similar to earlier ones to allow a better comparison between the classifications: We included only patients invasively mechanically ventilated in assisted-controlled or controlled pressure modes and those who collected arterial blood gases and had their ARDS severity classification established by the OI. Patients on noninvasive mechanical ventilation were not included. This makes it hard to compare our findings with earlier ones. Likewise, no studies have analyzed the correlation between the Berlin and PALICC classifications taking into account the presence or absence of bronchospasm and the use or absence of NMB.

The few studies that have compared the Berlin and PALICC classifications in pediatrics used broader inclusion criteria than the present study did, so they concluded that the PALICC criteria were superior the Berlin criteria.^(2.10)^

There are several limitations to our study. First, it was done in a single center. Although our unit can probably be compared to many other units in developing countries, our institutional practices may vary, such as the frequency of arterial blood gas analysis. Second, the evaluation of chest X-rays is subjective, as it has high interobserver variability.^(12)^ Third, and most importantly, our total sample was small due to our requirement of a 1-year period for data collection, which occurred amid the coronavirus disease 2019 pandemic, affecting the profile of patients admitted to the pediatric ICU. Moreover, the number of samples with bronchospasm decreased due to the change in the seasonality profile of respiratory diseases in pediatric patients.

## CONCLUSION

There was a strong correlation between the two classifications of acute respiratory distress syndrome severity (Berlin and PALICC) in the setting of our pediatric intensive care unit in the overall study population and in the studied subgroups.

This study, however, suggests that there is an effect of the use of neuromuscular blockers on the classifications of severity of acute respiratory distress syndrome and, perhaps, in these cases, the use of the ratio between partial pressure of oxygen and fraction of inspired oxygen for stratification of the severity of the syndrome was more appropriate.

More studies with more robust samples and considering the same situations are needed to compare the two classifications. Accurate severity stratification allows for the optimization of treatment and the use of available resources to improve the outcome and reduce mortality in pediatric patients with acute respiratory distress syndrome.

## References

[1] Ashbaugh DG, Bigelow DB, Petty TL, Levine BE (1967). Acute respiratory distress in adults. Lancet.

[2] Cheifetz IM (2017). Pediatric ARDS. Respir Care.

[3] Parvathaneni K, Belani S, Leung D, Newth CJ, Khemani RG (2017). Evaluating the Performance of the Pediatric Acute Lung Injury Consensus Conference Definition of Acute Respiratory Distress Syndrome. Pediatr Crit Care Med.

[4] Yehya N, Servaes S, Thomas NJ (2015). Characterizing degree of lung injury in pediatric acute respiratory distress syndrome. Crit Care Med.

[5] Wong JJ, Phan HP, Phumeetham S, Ong JS, Chor YK, Qian S, Samransamruajkit R, Anantasit N, Gan CS, Xu F, Sultana R, Loh TF, Lee JH, Pediatric Acute & Critical Care Medicine Asian Network (PACCMAN) (2017). Risk stratification in pediatric acute respiratory distress syndrome: a multicenter observational study. Crit Care Med.

[6] Sim J, Wright CC (2005). The kappa statistic in reliability studies: use, interpretation, and sample size requirements. Phys Ther.

[7] Mukaka MM (2012). Statistics corner: a guide to appropriate use of correlation coefficient in medical research. Malawi Med J.

[8] Sessler CN, Gosnell MS, Grap MJ, Brophy GM, O'Neal PV, Keane KA (2002). The Richmond Agitation-Sedation Scale: validity and reliability in adult intensive care unit patients. Am J Respir Crit Care Med.

[9] Wilsterman ME, de Jager P, Blokpoel R, Frerichs I, Dijkstra SK, Albers MJ (2016). Short-term effects of neuromuscular blockade on global and regional lung mechanics, oxygenation and ventilation in pediatric acute hypoxemic respiratory failure. Ann Intensive Care.

[10] Wittenstein J, Huhle R, Leiderman M, Möbius M, Braune A, Tauer S (2023). Effect of patient-ventilator asynchrony on lung and diaphragmatic injury in experimental acute respiratory distress syndrome in a porcine model. Br J Anaesth.

[11] Khemani RG, Smith L, Lopez-Fernandez YM, Kwok J, Morzov R, Klein MJ, Yehya N, Willson D, Kneyber MCJ, Lillie J, Fernandez A, Newth CJL, Jouvet P, Thomas NJ, Pediatric Acute Respiratory Distress Syndrome Incidence and Epidemiology (PARDIE) Investigators, Pediatric Acute Lung Injury and Sepsis Investigators (PALISI) Network (2019). Paediatric acute respiratory distress syndrome incidence and epidemiology (PARDIE): an international, observational study. Lancet Respir Med.

[12] Meade MO, Cook RJ, Guyatt GH, Groll R, Kachura JR, Bedard M (2000). Interobserver variation in interpreting chest radiographs for the diagnosis of acute respiratory distress syndrome. Am J Respir Crit Care Med.

