# Supplementary material for: Evaluation of the classifications of severity in acute respiratory distress syndrome in childhood by the Berlin Consensus and the Pediatric Acute Lung Injury Consensus Conference
Source: Crit Care Sci. 2024 May 20;39:e20240229en. doi: 10.62675/2965-2774.20240229-en (PMC11152442; doi:10.62675/2965-2774.20240229-en)
Supplement: Translation

# Translation: Avaliação das classificações de gravidade na síndrome do desconforto respiratório agudo na infância pelo Consenso de Berlim e pelo *Pediatric Acute Lung Injury Consensus Conference*

**DOI:** 10.62675/2965-2774.20240229-pt

**Authors:** Roberta Costa Capela, Raquel Belmino de Souza, Maria de Fátima Pombo Sant’Anna, Clemax Couto Sant’Anna

**Affiliations:** 1 Brasil Programa de Pós-Graduação em Saúde Materno-Infantil, Instituto de Puericultura e Pediatria Martagão Gesteira, Universidade Federal do Rio de Janeiro - Rio de Janeiro (RJ), Brasil.; 2 Brasil Unidade de Terapia Intensiva Pediátrica, Instituto de Puericultura e Pediatria Martagão Gesteira, Universidade Federal do Rio de Janeiro - Rio de Janeiro (RJ), Brasil.

**Keywords:** Índice de Gravidade de Doença, Síndrome do desconforto respiratório, Classificação de Berlim, Classificação PALICC, Espasmo brônquico, Bloqueadores neuromusculares, Criança

## Abstract

**Objetivo:**

Comparar dois métodos para definição e classificação de gravidade na síndrome do desconforto respiratório agudo pediátrica: a classificação de Berlim, que utiliza a relação entre pressão parcial de oxigênio e fração inspirada de oxigênio e a classificação do *Pediatric Acute Lung Injury Consensus Conference*, que utiliza o índice de oxigenação.

**Métodos:**

Estudo prospectivo com pacientes de 0 - 18 anos com diagnóstico de síndrome do desconforto respiratório agudo e ventilados mecanicamente de forma invasiva, que forneceram de uma a três amostras de gasometria arterial, totalizando 140 medidas válidas. Essas medidas foram avaliadas quanto à correlação pelo teste de Spearman e à concordância pelo coeficiente kappa entre as duas classificações, inicialmente usando a população geral do estudo e, depois, subdividindo-a em pacientes com e sem broncoespasmo e com e sem o uso do bloqueador neuromuscular. Também foi verificado o efeito desses dois fatores (broncoespasmo e bloqueador neuromuscular) de forma separada e conjunta sobre ambas as classificações por meio da análise de variância para dois fatores.

**Resultados:**

Na população geral, composta de 54 pacientes com idades de 0 - 18 anos, foi encontrada forte correlação negativa pelo teste de Spearman (ρ -0,91; p < 0,001) e forte concordância pelo coeficiente kappa (0,62; p < 0,001) na comparação entre Berlim e *Pediatric Acute Lung Injury Consensus Conference*. Nas populações com e sem broncoespasmo e com e sem o uso do bloqueador neuromuscular, os coeficientes de correlação mantiveram valores semelhantes aos da população geral. Entretanto, para os pacientes sem uso do bloqueador neuromuscular, houve maior concordância entre as classificações em relação aos pacientes com uso do bloqueador neuromuscular (kappa 0,67 *versus* 0,56 com p < 0,001 em ambos). Acrescenta-se ainda o efeito significativo do uso do bloqueador neuromuscular sobre a relação entre pressão parcial de oxigênio e fração inspirada de oxigênio (análise de variância; F: 12,9; p < 0,001) e o índice de oxigenação (análise de variância; F: 8,3; p = 0,004).

**Conclusão:**

Houve forte correlação e concordância entre as duas classificações na população geral e nos subgrupos estudados, entretanto, há efeito significativo do uso do bloqueador neuromuscular sobre as classificações de gravidade da síndrome do desconforto respiratório agudo.

## INTRODUÇÃO

A síndrome do desconforto respiratório agudo (SDRA) manifesta-se como inflamação pulmonar, edema alveolar e falência respiratória hipoxêmica. A fisiopatologia dessa síndrome clínica é caracterizada, em progressão, pelas fases inflamatória, proliferativa e fibrótica. Em 1967, foi elaborada a primeira descrição da SDRA por Ashbaugh et al.^(1)^

Embora as primeiras descrições da SDRA incluíssem crianças, considerações pediátricas não foram abordadas no *American-European Consensus Conference* (AECC) nem na definição de Berlim. Uma definição específica para SDRA pediátrica foi criada recentemente pelo *Pediatric Acute Lung Injury Consensus Conference* (PALICC).^(2)^

É importante frisar que as definições da SDRA baseadas em adultos podem não ser aplicáveis na pediatria por uma variedade de razões. Diferenças anatômicas e fisiológicas levam bebês e crianças a serem mais vulneráveis a um insulto respiratório grave quando comparados com adultos, necessitando potencialmente de um limiar menor para intervenção. Além disso, pacientes mais jovens têm uma demanda metabólica maior e uma reserva cardiopulmonar menor do que adolescentes e adultos.^(2)^ Aplicações prévias das definições baseadas em adultos na pediatria, com a necessidade de mensuração da oxigenação arterial, podem ter levado à prevalência subestimada da SDRA pediátrica, devido ao menor uso de linhas arteriais em bebês e crianças. Considerações especiais também são necessárias para otimizar o manejo da SDRA na heterogênea população pediátrica, que vai desde recém-nascidos a adolescentes.

Diferente da definição de Berlim (Tabela 1), o PALICC (Tabela 2) emprega: oximetria de pulso quando a pressão parcial de oxigênio (PaO_2_) não está disponível, índice de oxigenação (IO) ou índice de saturação de oxigênio (ISO) para estratificação de gravidade, em vez da relação entre pressão parcial de oxigênio e fração inspirada de oxigênio (PaO_2_/FiO_2_), e evidência de doença parenquimatosa pulmonar, em vez de infiltrados radiológicos bilaterais na radiografia de tórax. Centros únicos ou estudos regionais têm avaliado a definição do PALICC, mas seu desempenho em grandes amostras internacionais é desconhecida.^(3-5)^

**Tabela 1:** Definição de Berlim para a síndrome do desconforto respiratório agudo

Síndrome do desconforto respiratório agudo		
Tempo	Dentro de 7 dias do conhecimento do insulto ou nova piora dos sintomas respiratórios	
Imagem pulmonar*	Opacificações bilaterais não totalmente explicadas por colapso alveolar ou nódulos	
Origem do edema	Falência respiratória não explicada por falência cardíaca ou por sobrecarga hídrica. Necessidade de excluir edema hidrostático (ecocardiograma) se nenhum fator de risco estiver presente	
Oxigenação	Leve	200mmHg < PaO_2_/FiO_2_ ≤ 300mmHg com PEEP ou CPAP ≥ 5cmH_2_O
	Moderada	100mmHg < PaO_2_/FiO_2_ ≤ 200mmHg com PEEP ≥ 5cmH_2_O
	Grave	PaO_2_/FiO_2_ ≤ 100mmHg com PEEP ≥ 5cmH_2_O

Fonte: Modificado de: ARDS Definition Task Force; Ranieri VM, Rubenfeld GD, Thompson BT, Ferguson ND, Caldwell E, Fan E, et al. Acute respiratory distress syndrome: the Berlin Definition. JAMA. 2012;307(23):2526-33.

PaO2 - pressão parcial de oxigênio; FiO2 - fração inspirada de oxigênio; PEEP - pressão expiratória positiva final; CPAP - pressão contínua de vias aéreas. * Tomografia computadorizada ou radiografia de tórax.

**Tabela 2:** Definição PALICC para a síndrome do desconforto respiratório agudo em pediatria

**Síndrome do desconforto respiratório agudo pediátrica**					
Idade	Excluir pacientes com doença pulmonar perinatal				
Tempo	Dentro de 7 dias do insulto				
Origem do edema	Falência respiratória não explicada por falência cardíaca ou sobrecarga hídrica				
Imagem radiológica	Nova imagem pulmonar compatível com doença do parênquima pulmonar				
Oxigenação	Ventilação mecânica não invasiva		Ventilação mecânica invasiva		
	PARDS		Leve	Moderada	Grave
	*Full face bilevel* ou CPAP > 5cmH_2_O		4 ≤ IO < 8	8 ≤ IO < 16	IO ≥ 16
	PaO_2_/FiO_2_ < 300mmHg		5 ≤ ISO < 7,5	7,5 ≤ ISO < 12,3	ISO ≥ 12,3
	S/F ≤ 264mmHg				
**Grupos específicos**					
Doença cardíaca cianótica		Deterioração aguda a oxigenação não explicada pela doença de base			
Doença pulmonar crônica		Novo infiltrado e deterioração aguda da oxigenação de acordo com os critérios acima			
Disfunção ventricular esquerda		Novo infiltrado e deterioração aguda da oxigenação de acordo com os critérios acima não explicada por disfunção do ventrículo esquerdo			

Fonte: Modificado de Pediatric Acute Lung Injury Consensus Conference Group. Pediatric acute respiratory distress syndrome: consensus recommendations from the Pediatric Acute Lung Injury Consensus Conference. Pediatr Crit Care Med. 2015;16(5):428-39.

PARDS - síndrome do desconforto respiratório agudo em pediatria; CPAP - pressão contínua de vias aéreas; IO - índice de oxigenação; ISO - índice de saturação de oxigênio; PaO_2_ - pressão parcial de oxigênio; FiO_2_ - fração inspirada de oxigênio.

Na unidade de terapia intensiva (UTI) pediátrica do nosso serviço são utilizadas as duas classificações (Berlim e PALICC) para estratificação de gravidade da SDRA pediátrica desde 2015. O objetivo geral deste estudo foi classificar os pacientes internados na nossa UTI pediátrica com SDRA, segundo as duas classificações de gravidade (Berlim e PALICC) e os objetivos específicos foram descrever o perfil dos pacientes internados com SDRA no nosso serviço, comparar as duas classificações de gravidade e avaliar a influência da presença do broncoespasmo e do uso do bloqueador neuromuscular (BNM) nas classificações de gravidade da SDRA, na tentativa de estabelecer a classificação mais adequada da SDRA pediátrica para diagnóstico precoce e emprego direcionado dos recursos disponíveis.

## MÉTODOS

Tratou-se de estudo observacional prospectivo que foi realizado na UTI pediátrica do Instituto de Puericultura e Pediatria Martagão Gesteira da Universidade Federal do Rio de Janeiro (UFRJ) no período de dezembro de 2020 a dezembro de 2021. O instituto é um hospital pediátrico terciário que atende a população do Estado do Rio de Janeiro por meio do Sistema Único de Saúde (SUS). O estudo foi aprovado pelo Comitê de Ética em Pesquisa (CEP) do instituto (CAAE: 87266418.9.0000.5264).

Foram incluídos crianças e adolescentes de 0 - 18 anos com diagnóstico de SDRA e ventilados mecanicamente de forma invasiva nos modos pressóricos assisto-controlado ou controlado. Foram excluídos pacientes com sinais de hipertensão intracraniana, que preenchiam critérios para morte encefálica e/ou com instabilidade hemodinâmica, ventilados mecanicamente de forma invasiva no modo espontâneo e as amostras que deixaram de ter critérios diagnósticos para SDRA ao longo do período da coleta dos dados (3 dias). A amostra foi definida por conveniência, utilizando todos os pacientes internados na nossa UTI pediátrica que preenchiam os critérios acima no período predeterminado de 1 ano para a coleta de dados do estudo.

Os dados do prontuário foram coletados na admissão e ao longo de toda a internação do paciente na UTI pediátrica. Os dados à beira do leito (tanto do ventilador mecânico quanto da gasometria arterial) foram coletados em 3 dias ao longo da primeira semana do diagnóstico de SDRA, sendo a primeira coleta no dia da inclusão do paciente no estudo, a segunda coleta 24 horas após a inclusão e a terceira coleta 48 horas após a inclusão.

Foram calculados os índices de gravidade dos pacientes internados na UTI pediátrica: *Pediatric Index of Mortality* (PIM) na primeira hora da admissão e *Pediatric Risk of Mortality* (PRISM) nas primeiras 24 horas da admissão.

O uso de sedação para a coleta da gasometria arterial foi necessário em alguns casos, visto que pacientes sob ventilação assisto-controlada poderiam apresentar medidas de pressão de pico inspiratória variáveis, a depender de seu grau de agitação, não das características fisiopatológicas da doença.

O banco de dados foi construído no programa Microsoft Excel® versão 2013 (Microsoft Corporation, USA). A análise dos dados foi realizada com o programa *Statisitcal Package for Social Sciences* (SPSS, IBM Corporation), versão 28.0.0.0. Inicialmente, foi feita a análise descritiva dos dados. As variáveis qualitativas foram apresentadas como tabelas de frequência absoluta e/ou percentual, e as variáveis quantitativas foram apresentadas como medidas de tendência central e dispersão; média, erro padrão e mediana. Para as variáveis quantitativas, foi verificada a normalidade das amostras pelo teste de Kolmogorov-Smirnov, com nível de significância estatística ≥ 0,05.

Para as análises comparativas entre os dois critérios, Berlim (PaO_2_/FiO_2_) e da PALLIC (IO), verificamos a presença de concordância entre as classificações de gravidade pelo coeficiente kappa (κ). Foram considerados os seguintes valores para o coeficiente de concordância κ: concordância muito fraca de zero a 0,19; fraca de 0,2 a 0,39; moderada de 0,4 a 0,59; forte de 0,6 a 0,79 e muito forte ≥ 0,8.^(6)^ A correlação entre os valores dos dois índices (PaO_2_/FiO_2_ e IO) foi avaliada pelo teste de Spearman. Foram considerados os seguintes valores para o coeficiente de correlação de Spearman: entre zero e 0,3, correlações desprezíveis; entre 0,31 e 0,5, fracas; entre 0,51 e 0,7, moderadas; entre 0,71 e 0,9, fortes, e > 0,9, muito fortes, sejam seus valores positivos ou negativos.^(7)^ Para os dois testes, foi considerado nível de significância estatística ≤ 0,05.

Ainda nas análises comparativas, foram avaliados fatores que poderiam exercer influência sobre os critérios de classificação da SDRA. Assim, os pacientes foram separados em dois grupos: com e sem a presença de broncoespasmo/doença obstrutiva de vias aéreas inferiores e com e sem o uso do BNM, avaliando-se concordância e correlação pelos testes de κ e Spearman, considerando significância estatística ≤ 0,05.

Foi avaliada a influência das variáveis independentes broncoespasmo/doença obstrutiva de vias aéreas inferiores e BNM sobre Berlim e PALICC de forma separada e conjunta por meio do teste de variância (ANOVA) para dois fatores. Foi considerada significância estatística quando p ≤ 0,05. O mesmo procedimento foi realizado para algumas variáveis relacionadas à mecânica respiratória.

Nas situações em que foi realizada comparação entre médias e medianas, foram utilizados o teste T para amostras independentes e o teste de Man-Whitney, respectivamente, com significância estatística ≤ 0,05.

## RESULTADOS

Houve 56 pacientes internados com diagnóstico de SDRA e dois foram excluídos por negativa de consentimento.

Dentre os 54 pacientes do estudo, a mediana de idade foi de 30 meses, peso de 10kg, PIM2 de 5,6% e PRISM de 4,8%. Trinta de dois (59,3%) pacientes eram do sexo masculino, e 35 (64,8%) apresentavam comorbidades no momento do diagnóstico de SDRA. Entre os diagnósticos de internação, a pneumonia correspondeu a 34 (63%) casos, a bronquiolite a 4 (7,4%) e a leucemia linfoblástica aguda a 3 (5,6%). A SDRA de origem pulmonar foi responsável por 35 (64,8%) casos. A mediana de dias de internação foi de 12 dias, com tempo mínimo de 4 dias e máximo de 86 dias. A mediana de tempo de ventilação mecânica invasiva foi de 8 dias, variando de 3 a 66 dias. Pacientes com broncoespasmo corresponderam a 21 (38,9%).

Houve 14 óbitos (25,9%) no período do estudo. Apenas três doentes (5,5%) morreram no período de 3 dias da coleta de dados, tendo o restante dos óbitos ocorrido posteriormente ao longo da internação na UTI pediátrica. Os três óbitos iniciais foram de pacientes com comorbidades como imunodeficiência combinada, atresia de vias biliares e linfoma de Burkitt, tendo sido classificados como SDRA moderada evoluindo para grave. As causas dos óbitos foram choque, hipoxemia refratária e disfunção de múltiplos órgão e sistemas.

Ao longo dos 3 dias da coleta de dados, foram utilizados parâmetros ventilatórios semelhantes, com mediana de pressão expiratória positiva final (PEEP) de 8cmH_2_O, FiO_2_ de 60%, pressão inspiratória de pico (PIP) de 24cmH_2_O, volume corrente (Vc) de 8mL/kg, PaO_2_/FiO_2_ de 145 e IO de 11.

Os dados da ventilação mecânica dos doentes incluídos no estudo foram testados quanto à presença de normalidade da amostra pelo teste de Kolmogorov-Smirnov. Apenas a PaO_2_/FiO_2_ apresentou distribuição normal (p = 0,2).

Os 54 pacientes que participaram do estudo tiveram de uma a três amostras de gasometria arterial coletadas no período de 7 dias do diagnóstico de SDRA. Os que tiveram menos de três amostras coletadas tiveram suas amostras excluídas por perderem os critérios classificatórios para SDRA, evoluírem para óbito ou trocarem o modo ventilatório para espontâneo no curso desses 3 dias, permanecendo os dados até o momento anterior a sua exclusão.

Não houve critérios para SDRA segundo as duas classificações em 13 amostras (8%), segundo o PALICC em 4 amostras (2,5%) e segundo Berlim em 2 amostras (1,2%) ao longo dos 3 dias, totalizando 19 amostras; e ocorreram 3 óbitos nesse mesmo período da coleta de dados. Portanto, 22/162 (13,5%) amostras foram retiradas das análises comparativas posteriormente, restando 140 amostras válidas.

Em 104 (74,3%) medidas, os pacientes se encontravam em escala de agitação e sedação de Richmond (RASS)^(8)^ entre -1 e -5, em 69 (49,3%) os pacientes apresentavam disparo espontâneo, e em 135 (96,4%) havia sincronia paciente-ventilador mecânico. O uso do BNM esteve presente em 72 (51,4%) das 140 medidas, e em 90 (64,3%) não havia broncoespasmo associado. Foi realizada medida de mecânica ventilatória em 55 (39,2%) do total de medidas, sendo 42 (30%) medidas em uso de BNM. Encontramos medianas de complacência estática de 0,56mL.cmH_2_O^-1^.kg^-1^, pressão de platô (Pplat) de 25cmH_2_O e *driving pressure* de 14cmH_2_O.

Utilizando o universo de 140 amostras com diagnóstico de SDRA pelos dois critérios classificatórios ao longo dos 3 dias, foram obtidos os resultados apresentados na tabela 3.

**Tabela 3:** Pacientes com síndrome do desconforto respiratório agudo pelos dois critérios de classificação ao longo dos 3 dias e concordância entre as classificações

**Classificação de gravidade de SDRA**	**Consenso de Berlim**	**PALICC**	
Leve	25 (17,9)	40 (28,6)	
Moderada	75 (53,6)	57 (40,7)	
Grave	40 (28,6)	43 (30,7)	
Total	140 (100)	140 (100)	
**Classificação de gravidade de SDRA**	**Concordância entre as classificações de gravidade de Berlim e PALLIC**		
		**Kappa**	**Valor de p**
Leve	23 (16,4)	0,62	< 0,001
Moderada	49 (35,0)		
Grave	34 (24,3)		
Total	106 (75,7)		

SDRA - síndrome do desconforto respiratório agudo; PALICC - *Pediatric Acute Lung Injury Consensus Conference*.

Quando separadas as 50 amostras dos pacientes com broncoespasmo, houve concordância entre as duas classificações de SDRA em 38 medidas (76%); sendo 9 amostras (23,6%) para SDRA leve, 19 amostras (50%) para SDRA moderada e 10 amostras (26,3%) para SDRA grave [κ = 0,62 (p < 0,001)].

Para os pacientes sem broncoespasmo, que somaram 90 amostras, houve concordância entre as duas classificações de SDRA em 68 medidas (75,5%); 14 amostras (20,5%) para SDRA leve, 30 amostras (44,1%) para SDRA moderada e 24 amostras (35,2%) para SDRA grave [κ = 0,62 (p < 0,001)].

As amostras também foram comparadas quanto à concordância, de acordo com a utilização ou não do BNM como apresentado na tabela 4.

**Tabela 4:** Pacientes com síndrome do desconforto respiratório agudo pelos dois critérios de classificação, com e sem uso de bloqueador neuromuscular, ao longo dos 3 dias e concordância entre as classificações

**Classificação de gravidade de SDRA (SEM uso de BNM)**	**Consenso de Berlim**	**PALICC**	**Concordância**	
			**Kappa**	**Valor de p**
Leve	20 (29,4)	27 (39,7)	0,67	< 0,001
Moderada	34 (50,0)	27 (39,7)		
Grave	14 (20,6)	14 (20,6)		
Total	68 (100)	68 (100)		
**Classificação de gravidade de SDRA (COM uso de BNM)**	**Concordância entre as classificações de gravidadede Berlim e PALLIC**		**Concordância**	
			**Kappa**	**Valor de p**
Leve	5 (6,9)	13 (18,0)	0,56	< 0,001
Moderada	41 (56,9)	30 (41,7)		
Grave	26 (36,2)	29 (30,3)		
Total	72 (100)	72 (100)		

SDRA - síndrome do desconforto respiratório agudo; BNM - bloqueio neuromuscular; PALICC - *Pediatric Acute Lung Injury Consensus Conference*. Resultados expressos como n (%), quando não indicado de outra forma.

Em 60% das amostras com broncoespasmo, os pacientes estavam em uso de BNM. A concordância entre as classificações para essas amostras foi de 76,6%, com coeficiente κ = 0,59. Para as amostras com broncoespasmo sem o uso de BNM, a concordância entre as classificações foi semelhante: 75,5% com κ = 0,6. Os pacientes em uso de BNM sem broncoespasmo apresentaram concordância de 71%, com coeficiente de concordância K = 0,54. Já nas amostras em que não houve uso de BNM nem a presença de broncoespasmo, a concordância entre as classificações foi de 79%, com κ = 0,67. Para todos o nível de significância estatística foi ≤ 0,05.

Para a comparação entre os valores da PaO_2_/FiO_2_ e do IO, como mais uma forma de correlacionar as duas variáveis, foram utilizadas as 140 amostras válidas. Uma correlação negativa foi encontrada no teste de correlação de Spearman (ρ -0,918), com significância estatística (p < 0,001).

Os 50 pacientes com broncoespasmo apresentaram correlação de Spearman (ρ -0,91) e os 90 sem broncoespasmo, ρ -0,91. Os 72 pacientes que fizeram uso do BNM apresentaram correlação de Spearman (ρ -0,92) e os 68 que não fizeram uso do BNM, ρ -0,9. Todos esses dados apresentaram significância estatística com p < 0,001.

Foi realizada a Anova para dois fatores para avaliar o efeito das variáveis independentes broncoespasmo/obstrução de vias aéreas inferiores, BNM e os dois juntos sobre os critérios de classificação da SDRA (Berlim e PALICC), além de algumas outras variáveis relacionadas à mecânica respiratória (Tabela 5). As variáveis quantitativas envolvidas no teste da Anova apresentaram distribuição normal ou homogênea, verificadas pelo teste de Kolmogov-Smirnov e pelo teste de Levene, respectivamente, com significância estatística para ambos (p > 0,05).

**Tabela 5:** Comparação entre médias e medianas e influência da presença de bloqueador neuromuscular e/ou de obstrução de vias aéreas inferiores (broncoespasmo), nos pacientes com critérios para síndrome do desconforto respiratório agudo pelo *Pediatric Acute Lung Injury Consensus Conference* e pelo Consenso de Berlim

	BNM	n	Média ± DP	Mediana	Efeito BNM		Efeito obstrução VAI		Efeito BNM e obstrução VAI	
					Teste F	Valor de p	Teste F	Valor de p	Teste F	Valor de p
PaO_2_/FiO_2_	Sim	72	126,3 ± 52,8	126	12,9	< 0,001	0,029	0,86	7,36	0,39
	Não	68	161,7* ± 65,1	163,5						
IO	Sim	72	17,8 ± 15,7	13,4†	8,7	0,004	1,94	0,16	0,04	0,83
	Não	68	11,4 ± 8,0	8,7						
C.din (mL.cm H_2_O^-1^.kg^-1^)	Sim	72	0,37 ± 0,14	0,37	23,09	< 0,001	3,84	0,05	5,1	0,03
	Não	66	0,51 ± 0,25	0,48†						
C.est (mL.cm H_2_O^-1^.kg^-1^)	Sim	42	0,61 ± 0,36	0,55	0,001	0,98	0,54	0,46	0,61	0,44
	Não	13	0,62 ± 0,30	0,62‡						
PIP (cmH_2_O)	Sim	72	27,8 ± 5,2	27,0†	22,1	< 0,001	4,98	0,03	2,3	0,13
	Não	68	23,8 ± 6,3	22,0						
Vc	Sim	72	7,9 ± 3,5	8,0‡	0,3	0,58	0,71	0,40	0,004	0,95
	Não	68	7,6 ± 2,4	7,0						
MAP (cmH_2_O)	Sim	72	17 ± 3,7	16,5†	12,6	< 0,001	5,3	0,03	0,04	0,95
	Não	68	14 ± 3,8	13,8						

BNM - bloqueio neuromuscular; VAI - vias aéreas inferiores; PaO_2_ - pressão parcial de oxigênio; FiO_2_ - fração inspirada de oxigênio; IO - índice de oxigenação; Cdin - complacência dinâmica; Cest - complacência estática; PIP - pressão inspiratória de pico; Vc - volume corrente; MAP - pressão média de vias aéreas. * Diferença de médias - teste T - p < 0,05; † diferença de medianas - teste U - p < 0,05 ou ‡ p > 0,05.

## DISCUSSÃO

Neste estudo, foi possível observar que o perfil dos pacientes pediátricos internados na nossa UTI pediátrica foi compatível com o encontrado na literatura quanto à epidemiologia, morbidade e mortalidade na SDRA pediátrica.^(8-11)^

O principal achado deste estudo foi a observação de uma concordância significativa entre as classificações de gravidade da SDRA pediátrica (Berlim e PALICC), acompanhada por uma correlação numérica muito forte entre a PaO_2_/FiO_2_ e o IO e a presença de efeito significativo do BNM sobre essas classificações.

A presença de broncoespasmo, assim como a dissincronia paciente-ventilador, pode levar a um aumento da PIP devido ao aumento da pressão resistiva e da auto-PEEP e não da pressão elástica, sendo essa última relacionada com a fisiopatologia da SDRA. O aumento da PIP, leva a um aumento da pressão média de vias aéreas (MAP - *mean* airway *pressure*) e, com isso, ocorre o aumento no IO, que poderia ser interpretado como uma SDRA mais grave do que a real.

Nos pacientes em uso de BNM ou em sedação profunda com a supressão do disparo respiratório espontâneo, não há interferência do esforço do paciente na ventilação, sendo possível avaliar os dados da mecânica respiratória como complacência estática.^(9)^ Por outro lado, a supressão do *drive* respiratório e do esforço do doente, levam a aumento da pressão pleural, com necessidade de maior pressão de distensão e PEEP.

Portanto, as hipóteses secundárias de que a presença de broncoespasmo e/ou o uso do BNM poderiam exercer influência sobre os critérios de classificação da SDRA mereciam ser analisadas.

Neste estudo houve maior concordância entre as classificações nos pacientes sem uso do BNM, superior ao observado nos pacientes em uso do BNM, sugerindo influência dele nas classificações de gravidade da SDRA. Para pacientes com broncoespasmo, não foi observada tal diferença.

Entretanto, quando combinadas as quatro situações com e sem a presença de broncoespasmo/obstrução de vias aéreas inferiores e com e sem o uso do BNM, foi observada tendência de efeito de ambas as variáveis sobre as classificações da SDRA. Tais efeitos foram testados posteriormente pela Anova para dois fatores, com resultados mais expressivos para a variável BNM sobre a PaO_2_/FiO_2_ e o IO. Apesar de haver efeito significativo do broncoespasmo/obstrução de vias aéreas inferiores sobre a PIP, MAP e complacência dinâmica, não se observou efeito sobre a PaO_2_/FiO_2_ e o IO de forma independente ou em conjunto com a variável BNM.

Considerando o efeito significativo do uso do BNM sobre as classificações de gravidade (PaO_2_/FiO_2_ e IO) e sobre a PIP e a MAP (Figura 5) e os efeitos do seu uso em pacientes com SDRA, a supressão do *drive* respiratório ocasiona frequentemente a necessidade de aumento da pressão de distensão e da PEEP pelo aumento da pressão pleural, favorecendo maior colapso, principalmente nas regiões dependentes. Tais efeitos já foram descritos anteriormente em pacientes com sedação profunda e maximizados em pacientes em uso de BNM.^(9)^

O aumento das pressões necessário para compensar a ausência do esforço do paciente leva consequentemente ao aumento da MAP, que, por sua vez, leva ao aumento do IO, e, como resultado, o paciente é classificado com SDRA de gravidade maior, quando em uso do BNM. Tal fato leva a uma discussão sobre a real gravidade da SDRA quando avaliada na presença de esforço respiratório do paciente e o quanto isso pode “mascarar” a gravidade da SDRA e auxiliar em mecanismos de lesão pulmonar secundários.^(10)^

A SDRA é uma doença de acometimento do parênquima pulmonar, comprometendo o componente elástico pulmonar, e a PaO_2_/FiO_2_ é um dos marcadores mais importantes e sensíveis da alteração da oxigenação relacionado à síndrome. Vale ressaltar que a diferença entre as definições de Berlim e PALICC está na presença da MAP e, como esta pode ser mais elevada em pacientes que fazem uso do BNM para compensar a ausência do *drive* respiratório do paciente, talvez a classificação de gravidade da SDRA pela PaO_2_/FiO_2_ seja mais apropriada nesses casos.

No presente estudo, após a verificação do efeito do BNM sobre as classificações da SDRA, foi analisado o comportamento de algumas variáveis pela comparação entre medianas para as situações com e sem BNM, dentre elas as complacências dinâmicas (C.din = Vc/PIP - PEEP total) e estáticas (C.est = Vc/Pplat - PEEP total). Foi observada diferença com significância estatística apenas para complacências dinâmicas, sendo o valor da mediana maior em pacientes sem BNM. Tais achados corroboram a hipótese do efeito do esforço muscular sobre a PIP gerando menores pressões (considerando que haja sincronia paciente-ventilador, presente na maioria dos pacientes do estudo). No entanto, tal diferença não foi observada nas complacências estáticas, cujo cálculo prescinde do efeito do esforço respiratório, sendo utilizada a Pplat e, logo, avaliado somente o componente elástico pulmonar.

Vale ressaltar que, em ambas as situações, os volumes correntes não apresentaram diferenças significativas nas suas medianas, reforçando o efeito das pressões nos valores observados para as complacências. Da mesma forma, a distribuição de pacientes classificados como SDRA moderada a grave pelos dois critérios, em uso ou não de BNM, foi bem semelhante, dando mais robustez aos resultados descritos.

O PALICC foi criado para superar as limitações das definições de SDRA baseadas em adultos, incluindo as diferenças epidemiológicas e a necessidade de medidas invasivas de oxigenação. O PARDIE^(11)^ confirmou o melhor desempenho do PALICC em termos de identificação de novos casos de SDRA pediátrica e estratificação de risco. Entretanto, no PARDIE, menos da metade dos novos diagnósticos de SDRA pediátrica foi realizado por meio da medida invasiva da oxigenação. Em contraste, na nossa unidade, linhas arteriais são instaladas rotineiramente no início da ventilação mecânica para melhor monitorização hemodinâmica fazendo com que todos os novos diagnósticos de SDRA fossem feitos por meio da coleta da gasometria arterial.

Neste estudo, procuramos estabelecer equivalência entre as amostras para possibilitar melhor comparação entre as classificações, incluindo apenas os pacientes ventilados mecanicamente de forma invasiva nos modos pressóricos assisto-controlado ou controlado e aqueles que coletaram gasometria arterial e tiveram sua classificação de gravidade da SDRA estabelecida pelo IO. Não foram incluídos pacientes em ventilação mecânica não invasiva. Desse modo, comparações mais aprofundadas com os estudos existentes apresentam restrições. Da mesma forma, não foram encontrados, na literatura, estudos que analisassem a correlação entre as classificações de Berlim e PALICC levando em consideração a presença ou não de broncoespasmo e o uso ou não do BNM.

É importante considerar que os poucos estudos que compararam as classificações de Berlim e PALICC em pediatria utilizaram critérios de inclusão mais abrangentes que o presente estudo e, consequentemente, concluíram que havia uma superioridade dos critérios PALICC sobre os critérios de Berlim.^(2,10)^

Existem várias limitações a serem consideradas. Primeiro, nosso estudo foi desenvolvido em centro único. Embora nossa unidade possa provavelmente ser comparada a muitas outras unidades baseadas em países em desenvolvimento, nossas práticas institucionais podem variar como, por exemplo, a frequência da coleta das gasometrias arteriais. Segundo, a avaliação da radiografia de tórax é muito subjetiva e já provou haver grande variabilidade inter-observador.^(12)^ Terceiro, e mais importante, nossa amostra total foi pequena devido ao período determinado de 1 ano para coleta de dados, que se deu em meio à pandemia da doença pelo coronavírus 2019 (COVID-19), modificando o perfil de pacientes internados na UTI pediátrica e fazendo com que as amostras com broncoespasmo fossem menores pela alteração do perfil de sazonalidade das doenças respiratórias na pediatria.

## CONCLUSÃO

Houve forte correlação entre as duas classificações de gravidade da síndrome do desconforto respiratório agudo (Berlim e PALICC) no cenário da nossa unidade de terapia intensiva pediátrica, na população geral do estudo e nos subgrupos estudados.

Este estudo, entretanto, sugere que há um efeito do uso do bloqueador neuromuscular sobre as classificações de gravidade da síndrome do desconforto respiratório agudo e, talvez, nesses casos, a utilização da relação entre pressão parcial de oxigênio e fração inspirada de oxigênio para estratificação de gravidade da síndrome fosse mais adequada.

Mais estudos, contendo amostras mais robustas e considerando as mesmas situações, com o objetivo de comparar as duas classificações, são necessários. Uma estratificação de gravidade adequada possibilita uma otimização do tratamento e da utilização dos recursos disponíveis, a fim de melhorar o desfecho e reduzir a mortalidade da síndrome do desconforto respiratório agudo pediátrica
